# SMARCB1/INI1-Deficient Extrarenal Rhabdoid Tumor: A Case Report of a Rare and Aggressive Soft Tissue Sarcoma

**DOI:** 10.7759/cureus.8273

**Published:** 2020-05-25

**Authors:** Nathaniel A Parker, Ammar Al-Obaidi, Daniel Lalich, Jeremy M Deutsch

**Affiliations:** 1 Internal Medicine, University of Kansas School of Medicine, Wichita, USA; 2 Pathology, Wesley Medical Center, Wichita, USA; 3 Hematology and Oncology, Cancer Center of Kansas, Wichita, USA

**Keywords:** extrarenal rhabdoid tumor, malignant rhabdoid tumor, smarcb1/ini1-deficient tumor, adult soft tissue sarcoma, rare cancers, soft tissue sarcoma

## Abstract

Malignant SMARCB1/INI1-deficient extrarenal rhabdoid tumors are aggressive tumors that are extremely rare in adults. A 56-year-old male presented with the chief complaints of unilateral lower abdominal and pelvic pain. He underwent urgent surgical intervention and mass resection with tissue sampling. After pathology confirmed the diagnosis, systemic chemotherapy with vincristine, doxorubicin plus ifosfamide, and mesna was administered. Following treatment, he experienced a durable and long-lasting response to therapy for this aggressive and rare soft tissue sarcoma. To date, the patient remains in complete remission following the cessation of chemotherapy. Malignant SMARCB1/INI1-deficient extrarenal rhabdoid tumors are aggressive neoplasms that are extremely rare in adults. We report a rare case of such a tumor and review the literature for its molecular, clinical, and imaging features.

## Introduction

The tumor-suppressor gene SWItch/sucrose non‐fermentable-related (SWI/SNF) matrix-associated actin-dependent regulator of chromatin subfamily B member 1 (SMARCB1)/INI1 was first described in the malignant rhabdoid tumor (MRT) of infancy [1]. In children, MRT commonly occurs in the kidneys, soft tissue, and central nervous system [2]. SMARCB1/INI mutations have become a defining characteristic for not only MRT, but also any neoplasm with a disposition for rhabdoid cytomorphology [1]. Cases of SMARCB1/INI1 loss have been reported in various tumors with mesenchymal and epithelial origins [1-2]. As a result, rhabdoid tumors are a mixed polyphenotypic group with sometimes other overlapping immunohistochemical and histologic findings, which can make the diagnosis challenging [1]. The majority of SMARCB1/INI-deficient malignancies with a rhabdoid cytomorphology, in any age and location, are typically aggressive, and prognosis is often poor [1-3]. Here, we describe a rare case of a malignant SMARCB1/INI1-deficient extrarenal rhabdoid tumor in a middle-aged adult.

## Case presentation

A 56-year-old Caucasian male presented to his primary care provider with the chief complaint of painless left lower abdominal, inguinal, and scrotal swelling. Past medical history was only notable for chronic moderate-to-heavy chewing tobacco use. Family history was notable only for smoking tobacco-associated lung and bladder cancer in his father and mother, respectively, and prostate cancer in his maternal grandfather. This symptom started six months prior to presentation and had been gradually progressing. He denied any previous abdominopelvic trauma or hernia diagnosis. Vital signs, the remainder of the physical examination, and laboratory testing were primarily benign. A detailed physical exam was remarkable to the suprapubic area. Ultrasound of the pelvis was obtained. Sonographically, fluid collections were not sizeable enough to explain his symptoms. CT scans with contrast of the abdomen and pelvis were performed and showed a large soft tissue mass (approximately 12 cm x 12 cm x 23 cm) originating from the left testicle or scrotum that extended superiorly out of the scrotum and into the inguinal canal. From there, the soft tissue mass tracked along the left side of the abdominal and oblique muscles. There was no radiologic evidence of a fat plane between the musculature which was suspicious for direct invasion. Moreover, retroperitoneal adenopathy was not evident, and there were no obvious signs of metastases in the abdominopelvic cavity. Exploratory laparoscopy with left orchiectomy and mass debulking was performed in an effort for symptom management, tumor resection, and diagnosis. Operative notes illustrated that gross tumor invasion was evident in the skin, abdominal wall, and pubic bone.

**Figure 1:**
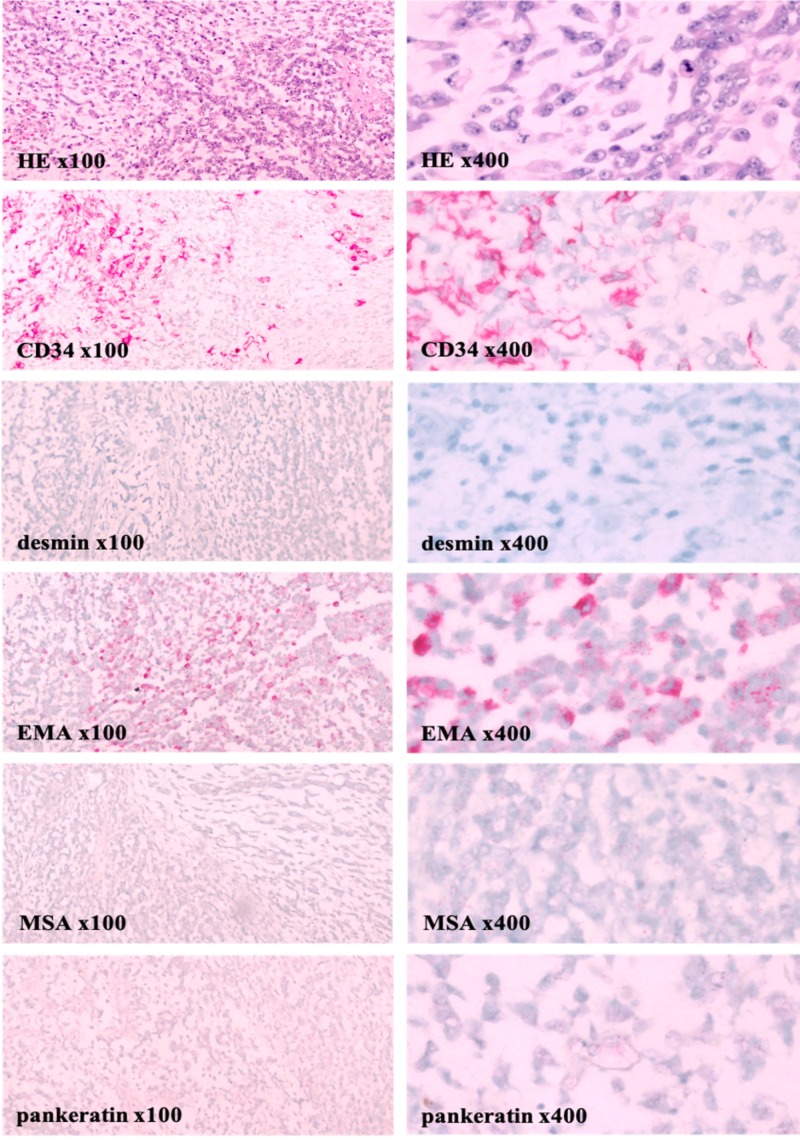
Left paratesticular tumor pathology demonstrates a sarcoma Tumor sectioning showed a uniform population of spindle-shaped tumor cells with prominent nucleoli and epithelioid tumor cells against a myxoid, cartilaginous-like stroma (HE x100 and x400). Mitotic rate was 8/10 high-power fields. Microscopically, no hypocellularity, fibrous areas, necrosis, or invasion in lymphovascular, vascular, or perineural tissue was identified. IHC stains on the malignant-appearing tissue were negative for pancytokeratin AE1/AE3 (pankeratin), S100, desmin, and muscle specific actin (MSA). Tissue specimens were partially immunoreactive to CD34 and epithelial membrane antigen (EMA) with approximately 30% of tumor cells being interpreted as positive. Thus, keratin, peripheral nerve, and smooth muscle-associated markers are negative. This IHC staining profile combined with features on light microscopy favored a high-grade retroperitoneal and paratesticular sarcoma. After additional review of the case by experts externally, the sarcoma was ultimately determined to be more likely a malignant extrarenal rhabdoid tumor.

Grossly, the ill-defined left inguinal/scrotal/testicular mass was 19 cm x 16 cm x 7 cm. Tumor invasion into paratesticular adipose tissue was apparent. Microscopically, sections of recognizable spermatic cord, epididymis, testis, and rete testis had no obvious evidence of primary malignancy such as a seminoma. Immunohistochemical (IHC) stains with proper controls were performed to define the tumor type. IHC stains on the malignant-appearing tissue were negative for pan-cytokeratin AE1/AE3 (pankeratin), S100, desmin, and muscle-specific actin (MSA). Tumor specimens were partially immunoreactive to CD34 and epithelial membrane antigen (EMA) markers (Figure 1). This IHC staining profile combined with histocytomorphologic features favored a high-grade retroperitoneal and paratesticular sarcoma. 

Three months following surgical debulking, contrasted CT scans of the chest, abdomen, and pelvis were obtained, which again showed no evidence of distant metastatic disease. However, residual disease was present and acute worsening was apparent based on radiologic evidence of new rapid growth. Progressive disease was observed in the left inguinal canal measuring 7 cm x 2.9 cm, a lower abdominal wall lesion measuring 16.6 cm x 10.4 cm, and one left inguinal lymph node measuring 2.5 cm. Subsequently the case was reviewed for a second opinion at an outside facility. There, a repeat CT of the abdomen and pelvis with contrast was obtained and confirmed progressive disease. A complex nodular mass in the subcutaneous tissues of the left inguinal region and abdominal wall was observed. However, its size had approximately doubled in all dimensions. Again, no evidence of metastatic disease was present. Additional testing was performed with revealed tumor cells, showing a loss of SMARCB1/INI1 expression and were FISH-negative for NR4A3. Ultimately, the patient’s tumor was determined to be more likely a malignant SMARCB1/INI1-deficient extrarenal rhabdoid tumor.

Due to the extent of disease, neoadjuvant chemotherapy was recommended. The patient underwent a regimen with vincristine, doxorubicin plus ifosfamide, and mesna. Supplemental dexrazoxane was added at the start of each chemotherapy cycle for cardioprotection. Prophylactic pegfilgrastim was given in between cycles for treatment-associated neutropenic fever and sepsis, which the patient was at an increased risk for developing on the basis of receiving myelosuppressive chemotherapy and the advanced stage of his disease.

Six months after the initiation of chemotherapy, a surveillance PET/CT showed complete resolution. This is consistent with a durable and long-lasting response to chemotherapy for this aggressive and rare soft tissue sarcoma. The patient remains alive, healthy, symptom-free, and in complete remission almost 12 months following cessation of chemotherapy.

## Discussion

Extrarenal rhabdoid sarcomas are rare soft tissue neoplasms in adults. Since being first described in 1970, its diagnosis is often difficult [4]. Loss of SMARCB1/INI expression has become a defining characteristic of malignancies with a rhabdoid cytomorphology since discovery of the SMARCB1/INI1 protein in the 1990s [2]. The diagnosis of these tumors is often difficult, partially owing to their mixed polyphenotype, as well as their retention of various parent tissue genotypic alterations and staining patterns [1-3]. Tissue biopsy is required to establish the final diagnosis, providing cytomorphologic and histopathologic evidence [3]. IHC can be used to identify inactivation of SMARCB1/INI1 and thereby support the diagnosis of malignant extrarenal rhabdoid tumors in adults [5].

In this case, the tumor was determined to be a high-grade neoplasm with rhabdoid morphology, most likely a malignant extrarenal rhabdoid tumor. Most rhabdoid tumors, regardless of location, are characterized by loss of expression of the SMARCB1/INI1 tumor-suppressor gene product. Keratin expression can be very limited in tissue samples. Also specimens may show expression of unusual markers, such as synaptophysin, a finding that was present in this case [6]. Due to some aspects of this lesion’s morphology, similarities can be seen between rare cases of extraskeletal myxoid chondrosarcoma, a lesion that can also be SMARCB1/INI1-deficient. Thus, FISH for NR4A3 rearrangement can be performed to help differentiate between the two neoplasms [6]. The diagnosis of extrarenal rhabdoid sarcomas, even after tissue collection, remains challenging. A SMARCB1/INI1-deficient phenotype can also be observed in certain poorly differentiated carcinomas [1-2]. However, none of these precursor lesions were seen in any of the patient’s specimens. Regarding the location of the patient’s soft tissue neoplasm, loss of SMARCB1/INI1 expression is not a feature of dedifferentiated liposarcoma, a tumor that frequently involves the paratesticular region. Ultimately, although it is difficult to be entirely dogmatic, the lesion was most likely a malignant extrarenal rhabdoid tumor, and was managed as such. Treatment of soft tissue sarcomas is yet to be standardized and generalized. Additional regimens may be added over time, as treatment for soft tissue sarcoma evolves. The most widely used treatment is referred to as AIM, a combined systemic chemotherapy regimen that consists of doxorubicin (Adriamycin) plus ifosfamide and mesna infusions [7]. Few therapeutic options currently exist for adult patients diagnosed with soft tissue sarcomas. The AIM regimen does provide hope for these patients, but much is still to learn. 

## Conclusions

Malignant SMARCB1/IN1-deficient extrarenal rhabdoid tumors are a variety of rare soft tissue sarcoma in adult patients with aggressive features. Tissue biopsy is the confirmatory test of choice. However, despite clinician due diligence and the presence of supportive histocytomorphologic evidence, the diagnosis remains challenging. IHC can help elucidate the inactivation of SMARCB1/INI1 and thereby support the diagnosis of malignant extrarenal rhabdoid tumors in adults. Early identification is imperative and helps guide therapy with chemotherapy regimens such as AIM.

## References

[1] Hollmann TJ, Hornick JL (2011). INI1-deficient tumors: diagnostic features and molecular genetics. Am J Surg Pathol.

[2] Kalimuthu SN, Chetty R (2016). Gene of the month: SMARCB1. J Clin Pathol.

[3] Margol AS, Judkins AR (2014). Pathology and diagnosis of SMARCB1-deficient tumors. Cancer Genet.

[4] Enzinger FM (1970). Epitheloid sarcoma: A sarcoma simulating a granuloma or a carcinoma. Cancer.

[5] Modena P, Lualdi E, Facchinetti F, Galli L, Teixeira MR, Pilotti S, Sozzi G (2005). SMARCB1/INI1 tumor suppressor gene is frequently inactivated in epithelioid sarcomas. Cancer Res.

[6] Oda Y, Tsuneyoshi M (2006). Extrarenal rhabdoid tumors of soft tissue: clinicopathological and molecular genetic review and distinction from other soft-tissue sarcomas with rhabdoid features. Pathol Int.

[7] Brenner T, Duggal S, Natale J (2020). UpToDate. Treatment protocols for soft tissue and bone sarcoma. https://www.uptodate.com/contents/treatment-protocols-for-soft-tissue-and-bone-sarcoma.

